# Factors Associated With Adolescents' Human Papillomavirus Vaccination Intention: A Cross‐Sectional Survey

**DOI:** 10.1002/nop2.70110

**Published:** 2024-12-08

**Authors:** Angela Chia‐Chen Chen, ChengChing Hiya Liu, Kimberly Arcoleo, Jiying Ling, Lorraine B. Robbins

**Affiliations:** ^1^ Michigan State University College of Nursing East Lansing Michigan USA

**Keywords:** adolescent, human papillowmavirus, intention, vaccination, youth

## Abstract

**Aim:**

To examine relationships between hypothesized potential predictors of vaccination and adolescent's Human papillomavirus (HPV) vaccination intention and whether these predictors differed by sex. We also investigated adolescents' experiences and preferences regarding learning about HPV through social media and other technology, to inform future tailored interventions.

**Design:**

Cross‐sectional research design.

**Methods:**

119 adolescents ages 11–17 years who had not received any HPV vaccine were enrolled. Purposive sampling was used to recruit participants from community and clinical settings, with the majority of households located in economically disadvantaged neighbourhoods in a southwest state. Participants completed an anonymous survey. Descriptive statistics, independent *t*‐tests, chi‐square test and hierarchical logistic regression were conducted to describe the sample, examine relationships between potential predictors and HPV vaccination intention and investigate sex differences.

**Results:**

The mean sample age was 13.76 years (SD = 2.03); 63% female; 42% Hispanic/Latino; 55.5% received free or reduced‐price lunch. Compared to White adolescents, Hispanic/Latino adolescents reported higher HPV vaccination intention (AOR = 24.10, CI 95% [3.85–150.82]; *p* < 0.001). Adolescents who had higher perceived risk of contracting HPV (AOR = 1.89, CI 95% [1.23–2.91]; *p* = 0.004) and perceived more facilitators (AOR = 1.61, CI 95% [1.22–2.12]; *p* < 0.001) reported higher HPV vaccination intention. Compared to boys, girls (AOR = 0.28, CI 95% [0.08–0.97]; *p* = 0.045) had lower HPV vaccination intention. Adolescents also showed strong interest in learning HPV‐related issues through digital education.

**Conclusion:**

Adolescent HPV vaccination rates in the United States are significantly below the targeted 80% completion goal. Nurses play a critical role in protecting adolescents from HPV infection and related cancers. Digital interventions addressing HPV vaccine‐related risks and facilitators and engaging key personnel (adolescent, parent, healthcare provider, teacher) in different settings have the potential to increase adolescent's vaccination intention.

**No Patient or Public Contribution to This Study:**

Patients or the general public were not involved in the design, analysis or interpretation of the data in this study.

## 1 Introduction

Human papillomavirus (HPV) is the most common sexually transmitted disease. In the United States, about 37,000 new cases of cancers (e.g., oropharyngeal, anal, cervical, vaginal) amongst males and females are diagnosed each year due to HPV (Centers for Disease Control and Prevention 2023a). Significantly, the annual deaths associated with HPV‐related cancers were estimated to be over 7000 with a substantial economic burden (US $3.8 billion) in the United States (Priyadarshini et al. 2020). Of the 14 million new HPV infections occurring each year; almost half occur in youth and young adults ages 15–24 years old (Meites et al. 2021), suggesting the importance of preventing HPV infection and its associated cancers in young populations.

HPV vaccination is the most effective strategy to prevent HPV infection and associated cancers and protection from it lasts a long time (Centers for Disease Control and Prevention 2021a). Routine HPV vaccination is recommended for boys and girls at age 11–12 years and can be initiated at age 9 (Centers for Disease Control and Prevention 2021b). Despite having a safe and effective vaccine to prevent HPV‐related cancers, only about 62.6% of children ages 13–17 years were up to date with their HPV vaccination (Pingali et al. 2023), indicating that the Healthy People 2030 target goal of 80% completion was not met (Office of Disease Prevention and Health Promotion 2020).

## 2 Background

HPV vaccination rates differ between adolescent boys and girls. While national statistics reported more adolescent girls were vaccinated against HPV than boys (Centers for Disease Control and Prevention 2023b), Barrett, Scoular, and Borgelt (2020) found no sex differences in the vaccination rates. Grandahl and Nevéus (2021) suggested the idea that HPV vaccination may increase promiscuity could contribute to lower vaccination in boys and young men compared to their female counterparts. The feminization of HPV and confusing recommendations for vaccination of males and females were also found to be associated with sexual disparities in vaccination rates (Daley et al. 2017). Regarding racial/ethnic differences, compared to their non‐Hispanic counterparts, Hispanic/Latino high‐school students reported higher HPV vaccination rates (Barrett, Scoular, and Borgelt 2020). HPV vaccination rates have been found the highest in girls living in economically disadvantaged, predominantly Hispanic communities (Henry et al. 2016). Other factors related to suboptimal HPV vaccination rates included low levels of knowledge and awareness of HPV and the vaccine in adolescents and their parents; parental negative attitudes towards vaccination; concerns about vaccine safety, side effects, effectiveness and cost; lack of recommendations from providers; nonmandatory vaccination and noncomprehensive insurance coverage (Zheng, Wu, and Zheng 2021). Other obstacles, such as perceived low risk of HPV infection (Chan et al. 2023), medical mistrust, limited access to healthcare, cultural beliefs and language barriers were also found to be related to adolescents' low HPV vaccination rates (Amboree and Darkoh 2021; Chen et al. 2017).

Despite growing efforts being directed towards promoting HPV vaccination amongst adolescents, the interventions have been primarily focused on parents (Bodson, Warner, and Kepka 2016; Galbraith et al. 2016) and healthcare providers (Dang et al. 2021) instead of the adolescents. Researchers (e.g., Pennella, Ayers, and Brandt 2020) have emphasised the importance of understanding adolescents' thoughts and feelings regarding HPV and the vaccine to inform the development of tailored and effective interventions for adolescents who are the vaccine recipients.

To assist with identifying antecedents of vaccination behaviours, Social Cognitive Theory (SCT) (Bandura 1986) and the Health Belief Model (HBM) (Rosenstock 1974, 1990), two widely applied theories in predicting vaccination behaviours (Li, Wood, and Kostkova 2022; Sacca et al. 2023) were used to guide this study. We examined the relationships between various potential predictors of vaccination (knowledge, perceived risk, perceived facilitators, perceived barriers, cultural beliefs) and adolescents' intentions to vaccinate against HPV and then investigated whether sex differences were observed in unvaccinated adolescents ages 11–17 years.

Digital devices (smartphone, computer/laptop, gaming consoles) and technology are widely accessible to adolescents: 93% of adolescents reported using YouTube and more than nine‐in‐ten adolescents in the United States used the internet daily (Pew Research Center 2023). Social media and other technologies could be effective educational channels to improve adolescents' understanding of HPV and its risks, benefits of HPV vaccination and available resources, leading to higher vaccination rates. Thus, we also explored adolescents' experience with social media and other technologies and their interests and preferred format for learning about HPV and the vaccine. The results will inform the development of an age‐ and culturally‐relevant digital intervention to enhance adolescents' intention to receive HPV vaccine, boost vaccination completion rates and prevent its related cancers.

## 3 The Study

### 3.1 Aims

This study aimed to examine relationships between hypothesised potential predictors of vaccination and adolescents' HPV vaccination intention and whether these predictors differed by sex. Because adolescents heavily use social media and other technologies for information, we also investigated adolescents' experiences and preferences regarding learning about HPV through social media and other technology, to inform future‐tailored interventions.

## 4 Methods

### 4.1 Design

This was a cross‐sectional, descriptive study utilising an anonymous self‐administered survey.

### 4.2 Sample, Sampling and Sample Size

An adolescent was eligible to participate if he or she (a) was aged 11–17 years, (b) had not yet received the first dose of the HPV vaccine, (c) could read and write in English or Spanish, and (d) provided parental consent and child assent. Purposive sampling was used to recruit a convenience sample from community and clinical settings, with the majority of households located in economically disadvantaged neighbourhoods in a southwest state of the United States. Power analyses were conducted using G*Power (Faul et al. 2007) specifying logistic regression, sample size = 119, df = 10, error probability = 0.05, and effect size *w* = 0.50. With the exception of biological sex (e.s. = 0.15), all effect size estimates were > 0.50.

### 4.3 Measures

All survey questions were pilot tested in prior research and found to be reliable and valid (Chen et al. 2017, 2021, 2019; Rambout et al. 2014; Lechuga, Swain, and Weinhardt 2011; Podolsky et al. 2009; Scarinci et al. 2010). The variables included in the survey were drawn from empirical evidence and theoretical constructs known to be associated with behavioural intention and changes. In general, values of Cronbach's alpha and KR‐20 are considered acceptable if they are 0.70 or above.

Sociodemographic characteristics and prior health education were assessed by 10 questions regarding age, biological sex, race and ethnicity, birthplace, parental education level, whether they received reduced‐price or free lunch in school and whether they received HPV education in the past and sources (if applicable).

The HPV knowledge scale included 12 true/false questions (e.g. both girls and boys can be infected with HPV) addressing known risk factors for HPV‐related infections and diseases and methods of detection. A sum score was computed, with a potential range of 0–12, with higher scores indicating higher levels of knowledge. The Kuder and Richardson Formula 20 (KR‐20), used to measure internal consistency reliability of a test with dichotomous items, was 0.67 in our sample. Perceived risks were measured by six true/false items (e.g. I don't have any physical symptoms or signs, so HPV vaccine is not needed) that assessed adolescents' perceived risk of being infected with HPV. A sum score (ranged from 0 to 6) was calculated with higher scores indicating greater perceived risk of contracting HPV infection. Cronbach's alpha (internal reliability) = 0.78 in this sample.

Perceived facilitators consisted of 10 yes/no items corresponding to the following factors promoting HPV vaccination behaviours: positive attitudes towards HPV vaccination (e.g. I want to do it to save my life), support from key personnel for vaccine decision‐making (e.g. If doctor or nurse tells me to do it) and resources (e.g., I will do it if my parents can afford it). A sum score was computed, with a potential range of 0–10. A higher score indicated a greater number of facilitators perceived by the adolescent. Cronbach's alpha = 0.80 in this sample. Perceived barriers included 15 yes/no items corresponding to a list of perceived barriers reported in previous research (Chen et al. 2017, 2021, 2019; Lechuga, Swain, and Weinhardt 2011; Podolsky et al. 2009; Scarinci et al. 2010) such as concerns about safety of the vaccine. A sum score was computed, with a potential range of 0–15, with higher scores indicating more barriers perceived by the adolescent. Cronbach's alpha = 0.80 in this sample.

Cultural beliefs about HPV were adapted from 3 items in the Cancer Screening Fatalism subscale of the Cultural Cancer Screening Scale (CCSS) (Betancourt et al. 2010), which had adequate reliability and predictive validity for cancer screening. The items (e.g. God will protect me, so HPV vaccine is not needed) had a 5‐point Likert‐type scale with response options ranging from 1 (*strongly disagree*) to 5 (*strongly agree*). The total score ranges from 3 to 15. A *lower* score indicated more favourable beliefs towards HPV vaccination. Cronbach's alpha = 0.70 in this sample. Social media use and preference for health education were assessed by 13 questions adapted from the literature (Pew Research Center 2023) to measure adolescents' experiences of, and preferences for, using social media (e.g. Twitter) and computerised programmes (e.g., video game) to learn about HPV and the vaccine.

### 4.4 Data Collection

We posted flyers in high‐traffic areas at communities and clinics. Trained research assistants contacted and screened the adolescents who contacted the team via QR code or in person and obtained parental consent and adolescent assent prior to participation. Adolescents received a survey link for a single anonymous survey administered via REDCap, a secure, web‐based application (Harris et al. 2009). The anonymous survey did not include any personal identifiers (e.g. name, social security number or address) or sensitive questions (e.g. sexual behaviour). Adolescents completed the survey in approximately 10–15 min. Each adolescent received a $10 gift card to acknowledge the time and effort needed to complete the survey.

### 4.5 Data Analysis

Data were imported into the Statistical Package for the Social Sciences (SPSS) 27.0 version (IBM Corp Released 2020) for analysis. We first conducted univariate analyses (e.g., mean, standard deviation, frequency, percentage) to describe distribution of study variables. The 2‐tailed independent *t* tests and chi‐square tests were used to examine differences in key variables by adolescent's biological sex. Bonferroni correction on multiple comparisons were not used due to the relatively small sample size. Hierarchical logistic regression investigated the relationship of hypothesised predictors (e.g. perceived risk, facilitators, barriers) with adolescents' vaccination intention adjusting for a priori covariates. We entered sociodemographic variables (i.e. age, sex, Hispanic/Latino, free/reduced‐price lunch) in the first block, followed by prior HPV information in the second block, with potential predictors of HPV vaccination in the third block.

### 4.6 Ethical Considerations

This study was approved by the University Institutional Review Board. Parental consent and adolescent assent were received prior to adolescents participating in the anonymous survey. The data were secured in a HIPAA‐compliant and encrypted server with multilevel password protection, enterprise‐level firewalls and antivirus barriers and could only be accessed by trained researchers.

## 5 Results

The sample included 119 adolescents ages 11–17 years [M (mean) = 13.76, SD (standard deviation) = 2.03]. Sixty‐three percent (*n* = 75) of the sample was female and 55.5% (*n* = 66) reported receiving free or reduced‐price lunches at school. Race/ethnicity were reported as: 42.0% Hispanic/Latino (*n* = 50), 16.8% non‐Hispanic white (*n* = 20), 15.1% non‐Hispanic black (*n* = 18), 13.4% non‐Hispanic Asian Americans (*n* = 16), 2.5% non‐Hispanic American Indians (*n* = 3) and 10.1% mixed race participants (*n* = 12). Only 18.5% (*n* = 22) of the sample reported receiving HPV vaccine information in the past. Amongst those who had received vaccine information, the sources included healthcare providers (40%), teachers (32%), families (24%) and friends (4%).

Regarding potential predictors of HPV vaccination, our sample had a moderately high knowledge score (*M* = 8.66, SD = 2.18), perceived relatively high risk of infection (*M* = 4.06, SD = 1.91), high level of facilitators (*M* = 6.94, SD = 2.52), low level of barriers (*M* = 4.20, SD = 3.30) and more favourable cultural beliefs towards vaccination (*M* = 6.44, SD = 2.56). Approximately 65% of the participants reported a positive intention to be vaccinated against HPV.

Specifically, the adolescents perceived some risk of contracting HPV infection due to their age and biological sex. Amongst the facilitators, the majority of participants indicated positive attitudes towards HPV vaccination (e.g. “I want to do it to save my life”), support from key personnel for vaccine decision‐making (e.g. “If parents tell me to do it”) and resources (e.g. “if I know where to get HPV vaccination”). For the barriers, participants identified concerns about vaccine safety and whether the vaccine worked. Participants also showed positive cultural beliefs towards HPV vaccination as suggested by low scores on the cultural belief items (e.g. “Being healthy or sick is my destiny. I cannot do anything to change it”).

### 5.1 Difference Between Boys and Girls

We compared the scores of the potential predictors by biological sex (Table 1). Amongst the five potential predictors associated with HPV vaccination intention, adolescent boys had significantly better knowledge scores (*M* = 9.18, SD = 2.03) than girls (*M* = 8.35, SD = 2.21; *t*_[117]_ = 2.048; *p* = 0.043). Boys also had higher perceived facilitator scores (*M* = 7.48, SD = 1.93) than girls (*M* = 6.22, SD = 2.77), but the difference was marginal (*t*_[116]_ = 1.970; *p* = 0.051). Compared to girls, a larger proportion of boys reported HPV vaccination intention (*χ* ^2^ = 8.952; *p* = 0.003). There were no significant differences in other potential predictors between adolescent boys and girls.

**TABLE 1:** Descriptive statistics and comparisons of theoretical factors between boys and girls.

Variable (score range)	Boy (*n* = 44)	Girl (*n* = 75)	Total (*n* = 119)	Effect sizes	*p*
	*M* (SD)	*M* (SD)	*M* (SD)		
HPV‐related knowledge (sum score, 0–12)	9.18 (2.03)	8.35 (2.21)	8.66 (2.18)	0.39	0.043*
Perceived risk (sum score, 0–6)	4.20 (1.68)	3.97 (2.04)	4.06 (1.91)	0.12	0.526
Perceived facilitator (sum score, 0–10)	7.48 (1.93)	6.22 (2.77)	6.94 (2.52)	0.53	0.051
Perceived barrier (sum score, 0–15)	4.36 (3.18)	4.11 (3.39)	4.20 (3.30)	0.08	0.686
HPV‐related cultural belief (sum score, 3–15)^a^	6.75 (2.88)	6.25 (2.34)	6.44 (2.56)	0.19	0.309

	*F* (%)	*F* (%)	*F* (%)		
Vaccination intention (yes)	36 (81.8%)	41 (54.7%)	77 (64.7%)	0.27	0.003*

^a^ Scale range 1–5. Lower scores indicate more favourable beliefs towards HPV vaccination.

* Results were statistically significant.

### 5.2 Hierarchical Logistic Regression Findings

Table 2 presents the results from the hierarchical logistic regression. In Model 1 where only the sociodemographic variables were included, a good model fit was obtained (*R* ^2^ = 0.30). In Model 2, the second block, adding prior HPV information, did not significantly contribute to the explanation of HPV vaccination intention (*R* ^2^ = 0.33). Model 3 containing all potential predictor variables was significant and accounted for 61% of the variance. Specifically, adolescents, who were Hispanic/Latino, had higher HPV vaccination intention scores compared to White adolescents [AOR = 24.10; 95%CI (3.85, 150.82); *p* < 0.001]. Compared to girls, boys had higher HPV vaccination intention [AOR = 0.28; 95%CI (0.08, 0.97); *p* = 0.045]. Adolescents who perceived greater risk [AOR = 1.89; 95%CI (1.23, 2.91); *p* = 0.004] and more facilitators [AOR = 1.61; 95%CI (1.22, 2.12); *p* < 0.001] had higher HPV vaccination intention scores.

**TABLE 2:** Hierarchical logistic regression analysis of relationship of theoretical factors with adolescent's vaccination intention.

Variables	Model 1		Model 2		Model 3	
	AOR (95% CI)	*p*	AOR (95% CI)	*p*	AOR (95% CI)	*p*
Age	1.25 (1.00–1.58)	0.052	1.23 (0.98–1.55)	0.076	0.91 (0.65–1.26)	0.560
Female	0.25 (0.09–0.68)	0.006*	0.24 (0.09–0.66)	0.006*	0.28 (0.08–0.97)	0.045*
Hispanic/Latino	5.93 (2.10–16.72)	< 0.001*	6.21 (2.14–17.99)	< 0.001*	24.10 (3.85–150.82)	< 0.001*
Free or reduce‐priced lunch at school	1.16 (0.45–3.00)	0.767	1.15 (0.43–3.06)	0.776	1.03 (0.30–3.53)	0.961
Prior HPV information			2.87 (0.80–10.38)	0.107	4.07 (0.83–19.89)	0.083
HPV‐related knowledge					1.06 (0.79–1.41)	0.693
Perceived risk					1.89 (1.23–2.91)	0.004*
Perceived facilitators					1.61 (1.22–2.12)	< 0.001*
Perceived barriers					0.96 (0.76–1.20)	0.702
HPV‐related cultural beliefs^a^					0.94 (0.72–1.23)	0.663
Model summary						
Nagelkerke *R* ^2^	0.302		0.328		0.606	
*χ* ^2^	28.63		31.51		67.02	
Df	4		5		10	
Model significance		< 0.001*		< 0.001*		< 0.001*

^a^ Scale range 1–5. Lower scores indicate more favourable beliefs towards HPV vaccination.

* Results were statistically significant.

### 5.3 Experiences and Preferred Format of Health Education

Regarding adolescents' experience with social media and other technologies and their preferred format for receiving HPV health education, 55.2% of the sample had experience using social media and 58.4% of them played computer games, mostly via smartphone and computer. Sixty‐four percent of the adolescents were interested in learning about HPV and the vaccine through social media, and 96.6% of them were interested in learning the content through health and educational games.

## 6 Discussion

This study examined hypothesized potential predictors (knowledge, perceived risk, perceived facilitators, perceived barriers, cultural beliefs) of HPV vaccination intention in unvaccinated adolescents ages 11–17 years and whether these predictors differed by biological sex. Consistent with prior research (Barrett, Scoular, and Borgelt 2020; Henry et al. 2016), Hispanic/Latino youth reported a higher HPV vaccination intention compared to their counterparts of other races/ethnicities. Also aligned with findings from a previously conducted study (Chan et al. 2023), adolescents were more willing to get the vaccine if they perceived a higher risk of contracting HPV.

Overall, we found a positive relationship between facilitators and vaccination intention in adolescents, consistent with prior research (Zheng, Wu, and Zheng 2021; Addisu, Gebeyehu, and Belachew 2023; Priest, Knowlden, and Sharma 2015; Oh et al. 2021). In this study, we assessed facilitators for vaccination by positive attitudes towards vaccination, support from key personnel for vaccine decision‐making, and available resources. We agree with other researchers (Priest, Knowlden, and Sharma 2015) about promoting positive attitudes towards vaccination by informing youth about the anticipatory benefits of HPV vaccination. Oh et al. (2021). found that parental approval and healthcare providers' recommendations were closely linked to youths' vaccine uptake. In this study, adolescents indicated the importance of teachers' support, in addition to parents and healthcare providers. This finding underscores the importance of engaging key stakeholders, including adolescent themselves, parents/guardians, healthcare providers and teachers in the collective efforts to prevent cancers through HPV vaccination. Furthermore, the adolescents identified other facilitators, including resources that helped them with vaccine decision‐making, such as vaccine affordability and awareness of where to receive HPV vaccine. This finding suggests the importance of incorporating practical information such as cost and vaccination locations to increase adolescents' intentions of getting it.

Findings from prior research have shown mixed relationships between HPV knowledge and vaccination intention or behaviour (vaccine uptake), with many of them suggesting no direct relationship between knowledge and either outcome. For instance, Fishman, Taylor, and Frank (2014) did not find any relationship between HPV knowledge and subsequent vaccination amongst U.S. adolescents. On the contrary, Woldehawaryat, Geremew, and Asmamaw (2023) reported a positive relationship between knowledge and vaccine uptake amongst Ethiopian adolescents. In the current study, adolescents had a high knowledge score; however, knowledge level was not significantly associated with vaccination intention while holding other variables constant. This finding suggests that knowledge alone may not be sufficient to influence adolescents' intention to get vaccinated. Instead, the impact of knowledge on vaccination intention and behaviour may occur indirectly through changes in beliefs, perceived risk, facilitators or barriers. Furthermore, the reliability of the knowledge scale in this sample was not optimal which may contribute to its non‐significant relationship with vaccination intention. Evidence indicates that individuals who perceived more barriers were less likely to be vaccinated (Donadiki et al. 2014). Perceived barriers, however, were not significantly associated with adolescents' vaccination intention in this study. This inconsistency could potentially be due to the relatively few barriers reported in our sample. Furthermore, our sample disagreed with the cultural beliefs about HPV. Since these items were adapted from the Cancer Screening Fatalism subscale of CCSS (Betancourt et al. 2010), they may not be applicable to adolescents in the vaccination context.

While national statistics and empirical evidence consistently reported higher HPV vaccination rates in girls than in boys, adolescent boys in our study reported a higher HPV vaccination intention than girls. This inconsistency may have resulted from different outcomes (vaccine uptake vs. intention) and sample characteristics (e.g. socioeconomic status, racial/ethnic distribution). Nevertheless, it is critical to inform the public about the importance of preventing HPV‐related diseases and cancers in both boys and girls. Similar to other research (Radovic et al. 2018), the vast majority of our adolescent participants showed strong interest in learning about HPV and the vaccine through digital technologies and platforms (video games, social media). Thus, digital interventions that can help adolescents accurately assess the risks of contracting infection (e.g. both boys and girls can be infected) and address facilitators (positive attitudes towards vaccination, support from key personnel for vaccine decision‐making, available resources) may increase their intention for vaccination.

Adolescents are digital natives and use social media and other technologies on a daily basis (Pew Research Center 2023). Social media and other technologies could be used as effective means to increase adolescents' health literacy, combat misinformation, provide timely resources and bridge health disparities through accessible and accurate health information to adolescents who may not be reached through traditional health education. Digital prevention interventions are cost‐effective approaches to promote HPV vaccination and prevent its associated morbidity and mortality in adolescents. Key personnel, including parents, public health nurses, other healthcare providers and teachers, are in ideal positions to coordinate essential resources and implement evidence‐based HPV prevention interventions tailored to adolescents' cultural, linguistic and developmental needs. Particularly, vaccination events held at schools will help remove some structural and logistical barriers such as cost, care access, transportation and scheduling issues. Community‐ and school‐based collaborative efforts to promote positive attitudes towards vaccination; provide support and recommendations from parents, nurses and other healthcare providers, and teachers for vaccination; and connect families with essential resources (e.g. community‐ and school‐based health centers) through digital platforms will allow for rapid and wide dissemination.

### 6.1 Strengths and Limitations

This study is unique as the findings were directly generated from adolescents, the HPV vaccine recipients. The anonymous nature of the survey should have reduced social desirability and consequently increased the validity of the data. Several limitations should be acknowledged. It is challenging to identify causal relationships between variables in cross‐sectional research. Furthermore, caution must be used when generalising the findings from this non‐representative sample with a relatively small sample size to other adolescent populations. The relatively low reliability of the knowledge items and cultural beliefs measures adapted from CCSS may not capture the true relationship of these factors with adolescents' HPV vaccination intention. Finally, HPV vaccine uptake was not assessed to examine its relationship with intention as we targeted unvaccinated adolescents.

### 6.2 Recommendations for Future Research

Future longitudinal research that includes larger and more diverse samples from broader geographic areas (e.g. urban vs. rural areas) and assesses adolescents' HPV vaccine uptake will enhance our understanding about appropriate health promotion strategies to address their unique needs.

## 7 Conclusion

According to adolescents' own voices, those who perceived a higher risk of contracting HPV and more facilitators (positive attitudes towards vaccination, support from key personnel for vaccine decision‐making, available resources) demonstrated a higher intention to be vaccinated against HPV and the related cancers, highlighting the need to address these aspects in HPV education. HPV education designed and delivered by digital technologies and platforms may be a more effective and promising approach for increasing vaccination rates in the adolescent populations.

## Author Contributions

Angela Chia‐Chen Chen contributed to study design and administration, data analysis, data interpretation and manuscript writing. All other authors contributed to data interpretation and manuscript writing. All authors read and approved the final draft of the manuscript. Dr. Kimberly Arcoleo and Dr. Jiying Ling are the statisticians on the author team.

## Conflicts of Interest

The authors declare no conflicts of interest.

## Data Availability

The authors have nothing to report.
